# Dicer increases the indication for trastuzumab treatment in gastric cancer patients via overexpression of human epidermal growth factor receptor 2

**DOI:** 10.1038/s41598-021-86485-8

**Published:** 2021-03-26

**Authors:** Jianhua Wu, Qun Zhao, Yue Zhao, Xiaoyun Zhang, Yuan Tian, Zhanjun Guo

**Affiliations:** 1 Animal Center, The Fourth Hospital of Hebei Medical University, Shijiazhuang, People’s Republic of China; 2 Department of Surgery, The Fourth Hospital of Hebei Medical University, Shijiazhuang, People’s Republic of China; 3 Department of Gastroenterology and Hepatology, The Fourth Hospital of Hebei Medical University, Shijiazhuang, People’s Republic of China; 4 Department of Immunology and Rheumatology, The Fourth Hospital of Hebei Medical University, 12 Jiankang Road, Shijiazhuang, 050011 People’s Republic of China

**Keywords:** Cancer, Cell biology, Molecular biology, Biomarkers, Gastroenterology

## Abstract

The low proportion of gastric cancer (GC) patients with high HER2 expression limits the clinical application of trastuzumab, a humanized epidermal growth factor receptor 2 (HER2) antibody targeting for GC treatment. We found that Dicer was positively correlated with HER2 expression in GC tissue by immunostaining as well as induce HER2 overexpression without increasing invasiveness of GC cell. In addition, both the growth of GC referring to cell proliferation, invasion, migration and apoptosis was inhibited by Dicer overexpression. Moreover, the HER2 overexpression induced by Dicer provided more effective and additive target for trastuzumab to amplify the inhibition effect for GC cells in vitro and in vivo. Furthermore, as assessed in a subsequent experiment, calcitriol induced HER2 overexpression and amplified the inhibition effect of trastuzumab in GC cells referring to proliferation. Our finding demonstrated the calcitriol might increase indication of trastuzumab by inducing HER2 overexpression in GC patients. Dicer would be a potential target that extend the clinical indications of HER2 antibody in patients with low or negative HER2, who were not fit for HER2 antibody treatment before.

## Introduction

Gastric cancer (GC) is the fourth most common cancer and the second leading cause of cancer mortality worldwide. One million cases are diagnosed annually worldwide, with approximately 50% of these found in China^1,2^. Most patients present with inoperable advanced or metastatic disease that has a 5-year survival rate of 5%–20% with median overall survival less than 1 year ^3^. The most popular treatment for this cancer is the fluoropyrimidine-based and platinum-based combination with or without a third drug such as docetaxel and epirubicin. Vascular endothelial growth factor (VEGF) and its receptor mediated signaling and angiogenesis play pivotal role in pathogenesis of GC; hence, various antiangiogenic agents (AAs) including both antibodies and tyrosine kinase inhibitors (TKIs) which target the angiogenic signaling pathways have been tested for GC treatment^3–7^, but only two AAs have been proved to improve the overall survival in GC or gastroesophageal junction (GEJ) cancer patients. Ramucirumab (VEGFR-2 antibody) is recommended as the second line treatment combined with panclitaxel or the third line treatment with monotherapy, whereas apatinib is recommended as the third line treatment with monotherapy^4–7^. The human epidermal growth factor receptor 2 (HER2) antibody trastuzumab could be used as the first line treatment combined with chemotherapy, and displays dramatically improved survival in GC and GEJ patients with high HER2 expression (median OS: 16 months vs. 11.8 months)^3^. HER2 is a member of the HER family (including HER-1, HER-3, and HER4) that is associated with tumor cell proliferation, apoptosis, adhesion, migration, and differentiation, and it encodes a 185-kDa plasma membrane-bound tyrosine kinase receptor on chromosome 17^8,9^. Increasing evidences reveal that HER2 triggers tumorigenesis associated with poor outcome and aggressive disease in GC^8,10^. Trastuzumab is a humanized monoclonal antibody that targets HER2 to inhibit HER2-mediated signaling transduction, and is widely used in metastatic breast cancer and advanced GC patients with high HER2 expression levels to achieve survival advantage^3,11,12^; however, the fact that only 7%–34% GC tissue exhibits high HER2 expression limited trastuzumab use in GC patients^3,8,13^.

MiRNAs are small noncoding RNAs of 18–25 nucleotides in length that bind to the 3′-untranslated region (3′UTR) of target mRNAs to repress targeted gene translation, thereby resulting in decreased encoded protein^14,15^. The cytoplasmic RNase III endonuclease Dicer, which cleaves the loop of the pri-miRNA and long double-stranded RNA into ~ 22 bp double-stranded miRNA and short interfering RNA (siRNA), is implicated in both regulation of cell proliferation and apoptosis control^16,17^. Dicer expression is associated with poor prognosis as a tumor suppressor in tumors including GC, hepatocellular carcinoma, and breast cancer^18–20^. Calcitriol is one of the important active metabolite of vitamin D that functions as a potent steroid hormone. Moreover, apart from calcium homeostasis and bone mineralization, calcitriol exhibits antitumor/anti-inflammatory effects in various cancers by regulating multiple signaling pathways involved in cell proliferation, apoptosis, differentiation, inflammation, invasion, angiogenesis, and metastasis^21–23^. Furthermore, calcitriol can induce Dicer expression in cervical cancer^24^. Since the Dicer expression was positively correlated with HER2 status in breast cancer^25^, we assessed the relationship between Dicer and HER2 in GC as well as their interaction with GC progression in the present study.

## Methods

### Human tissue specimens and immunohistochemistry

Gastric cancer tumor tissues were collected from 116 GC patients who underwent curative resection between June 2011 and December 2013 in the Department of General Surgery of the Fourth Hospital of Hebei Medical University. All procedures were supervised and approved by the hospital’s Human Tissue Research Committee. Written consent was obtained from all patients enrolled in this study.

Immunohistochemical analysis for Dicer was performed as described in a previous study using the streptavidin-peroxidase (SP) method^26^. After fixation with 10% formalin, the paraffin-embedded tumor tissues were cut into 4-μm thick sections and were stained with primary antibody against Dicer (1: 100 dilution, Abcam, Cambridge, UK) overnight at 4 °C, followed by incubation with a biotinylated secondary anti-mouse IgG antibody for 1 h at room temperature. Thereafter, the sections were subsequently incubated with HRP-conjugated streptavidin and were developed using 3, 3′-diaminobenzidine (DAB). The stained slides were scored using the HSCORE by two pathologists with knowledge of the clinical data of patients, according to the HSCORE as described previously (HSCORE = (i + 1) π. i = 1, 2, 3 and 4; i was weighted by the staining intensity as follows: 0, no staining; 1, weak staining, light-yellow; 2, moderate-weak staining, yellow brown; 3, moderate-strong staining, brown; and 4, strong staining, dark brown. Additionally, π was the sum of each of the percentages, ranging from 0 to 100%). A score of > 100 was defined as high expression and ≤ 100 as low expression^27^. HER2 immunohistochemical staining in GC tissue was performed with the fully automated BenchMark ULTRA platform (Ventana Medical Systems, Inc., Tucson, AZ) using the Pathway anti-HER2/neu (4B5) anti-body (Ventana Medical Systems, Inc., Tucson, AZ). HER2 expression was graded using a score of 0 to 3 + by two pathologists, with the scoring methods described previously^13^.

### Cell lines and cell culture

The Dulbecco’s Modified Eagle Medium (DMEM) high-glucose medium (Gibco Life Technologies, Grand Island, NY) or RPMI 1640 medium (Gibco Life Technologies) with 10% fetal bovine serum (FBS) (Gibco Life Technologies) was used for human GC cell lines culturing. The cell line of SGC7901 (RRID:CVCL_0520) and MGC803 (RRID:CVCL_5334) provided by Shanghai Institute of Biological Sciences and Cell Biology, Chinese Academy of Sciences (Shanghai, China) were incubated at 37 °C with a humidified incubator containing 5% CO2. Trastuzumab (Herceptin, Roche Pharma, South San Francisco, CA) with final concentration of 10 μg/mL was added into the medium for subsequent analysis.

### Cell transfection

Green fluorescent protein (GFP)-tagged Dicer-overexpressing lentivirus (pCMV-Dicer) and negative control lentivirus (pCMV-NC) were constructed by GeneCopoeia Corp (Rockville, MD). The SGC7901 and MGC803 cells were infected with lentivirus, according to the manufacturer’s instruction. To overexpress HER2, the coding sequence of HER2 was amplified and subcloned into the pEZ-Lv105 vector with a puromycin selection marker (GeneCopoeia, Rockville, MD). Thereafter, the MGC803 cells were transfected with HER2-pEZ-Lv105 and pEZ-Lv105 plasmids using lipofectamine 3000 (Invitrogen, San Diego, CA). Moreover, polyclonal populations of stably transfected cells were isolated by puromycin selection. Successful overexpression of Dicer and HER2 was confirmed by western blotting analysis.

### Western blotting analysis

The SGC7901 and MGC803 cells were lysed with radioimmunoprecipitation assay buffer containing protease inhibitor (Roche, Basel, Switzerland). Proteins from each sample were subjected to sodium dodecyl sulphate polyacrylamide gel electrophoresis (SDS-PAGE), and transferred onto polyvinylidene difluoride (PVDF) membranes (Roche, Basel, Switzerland). After blocking with 5% skim milk, the membrane was incubated with primary antibody against Dicer (dilution rate 1:1,000; Abcam, Cambridge, UK), HER2 (dilution rate 1:1,000; Abcam, Cambridge, UK), and β-actin (1:10,000 dilution; Santa Cruz, CA) at 4 °C overnight, followed by incubation with a HRP-conjugated anti-mouse IgG secondary antibody (1:5,000 dilution; Thermo Fisher, New York, NY) for 1 h at room temperature. The relative intensities of protein bands were visualized using an enhanced chemiluminescence reagent (Thermo Fisher, New York, NY) and analyzed using an FluorChem HD2 protein imprinting imaging system (Alpha Innotec, San Leandro, CA).

### Cell proliferation assay

Cell proliferation assay was measured with Cell Counting kit-8 (CCK-8; Dojindo, Kumamoto, Japan). Briefly, 96-well plates with six duplicate wells for each group was used for cell culturing, the GC cell with quantity of 10^3^ cells in each well was incubated at 37 °C with 10 µl CCK-8 in a humidified incubator containing 5% CO2 for 2 h. After that, the absorbance of each cell was calculated with a microplate reader (Bio-Tek, Winooski, VT) at wavelength of 450 nm for the different time points (0, 12, 24, 48, and 72 h).

### Wound healing assay

Cells were seeded on 6-well plates and cultured to approximately 100% confluence. A straight wound was drawn with a 200 μL pipette tip on the monolayer. After washing twice with PBS, the cells were further cultured in fresh medium with 2% FBS. Wound closure was monitored at 0, 12, and 24 h via inverted microscopy (Nikon, Tokyo, Japan). Migration rates were calculated as the width of a scratch divided by the initial width of the same scratch, as previously described^28^. All measurements were repeated thrice.

### Cell invasion assays

Cell invasion assays were performed in a 24-well transwell chamber with 8-µm pore size (Corning, New York, NY) precoated with 1 mg/mL BD Matrigel (BD Biosciences, Franklin Lakes, NJ). Cells were suspended in serum-free medium and seeded into the upper chamber at 1 × 10^5^/well, whereas the lower chamber was filled with 500 μL medium including 10% FBS. After incubation at 37 °C for 24 h, cells on the upper surface of the membrane were removed, and then the cells invaded into the underside were fixed with 4% paraformaldehyde and stained with 0.5% crystal violet. Stained cells were enumerated under an inverted microscope in 5 random fields of each membrane (magnification, × 200).

### Flow cytometry

Cell apoptosis was measured using PE Annexin V Apoptosis Detection Kit (BD Biosciences Pharmingen, San Diego, CA), following the manufacturer’s instructions. Each group of cells was harvested and washed twice with cold PBS, and thereafter, the cells were resuspended in the binding buffer. Subsequently, cells were incubated with 5 µL PE annexin V and 5 µL 7-aminoactinomycin D for 15 min in the dark at room temperature, and 400 μL binding buffer was added to each sample. The percentage of apoptotic cells was analyzed via flow cytometry using FACS Aria II flow cytometer (BD Biosciences) within 1 h. The experiments were repeated at least thrice.

### In vivo tumor growth assay

The protocol of animal experiment in this study was approved by Animal Ethnics Committee of The Fourth Hospital of Hebei Medical University. Sixty four-week-old male athymic nude mice of the BALB/c strain were purchased from Charles River Laboratories (Beijing, China; permission no. SCXK (Jing) 2016-0006). Nude mice were housed and treated in accordance with the guidelines established by the National Institutes of Health Guide for the Care and Use of Laboratory Animals^29^.

The SGC7901 and MGC803 cells treated with Dicer-lenti and NC-lenti were harvested and resuspended in PBS. Subsequently, groups of nude mice were subcutaneously injected at the shoulder with 0.2 mL of the cell suspensions (5 × 10^6^ cells/mouse). Moreover, mice were intraperitoneally injected with 15 mg/kg trastuzumab once every 3 days for 3 weeks since the 7th day^30^. After initiating trastuzumab administration, the tumor growth was monitored by measuring the tumor dimensions with digital calipers every 7 days, and the tumor volumes were calculated by the formula: Volume = Length × (Width)^2^/2. Furthermore, in vivo green fluorescent images were captured by NightOwl Bioimager (Berthold Technologies, Bad Wildbad, Germany) at 15 and 25 days after administration. The fluorescent intensity was analyzed by WinLight32 software package (Berthold Technologies).

### miRNA microarray analysis

The microarray experiment was commissioned to CapitalBio Corporation (Beijing, China). In brief, three samples were randomly selected from each group of pCMV-Dicer and pCMV transfected MGC803 cells, and total RNA was extracted and purified using miRNeasy Mini Kit (Qiagen, Valencia, CA), according to the manufacturer’s instructions. Microarray of miRNA was manufactured and processed as described^31^. The microarrays were scanned on GeneChip Scanner 3000 (Affymetrix, Santa Clara, CA), and the data were analyzed using a GeneChip Command Console software (Affymetrix).

### Statistical analysis

The data are reported as the mean value ± SD. Correlation between Dicer protein level and HER2 protein level was estimated using chi-square test. Cell proliferation, migration, invasion, apoptosis, and tumor growth assay were compared by one-way analysis of variance (ANOVA). Values of *p* < 0.05 were considered as statistically significant. Data was obtained from at least three independent experiments with a similar pattern. All statistical analyses were carried out using SPSS statistical software, version 19.0 (IBM Corporation, Armonk, NY).

## Results

### HER2 overexpression was mediated by dicer in gastric cancer tissue

**Figure 1:**
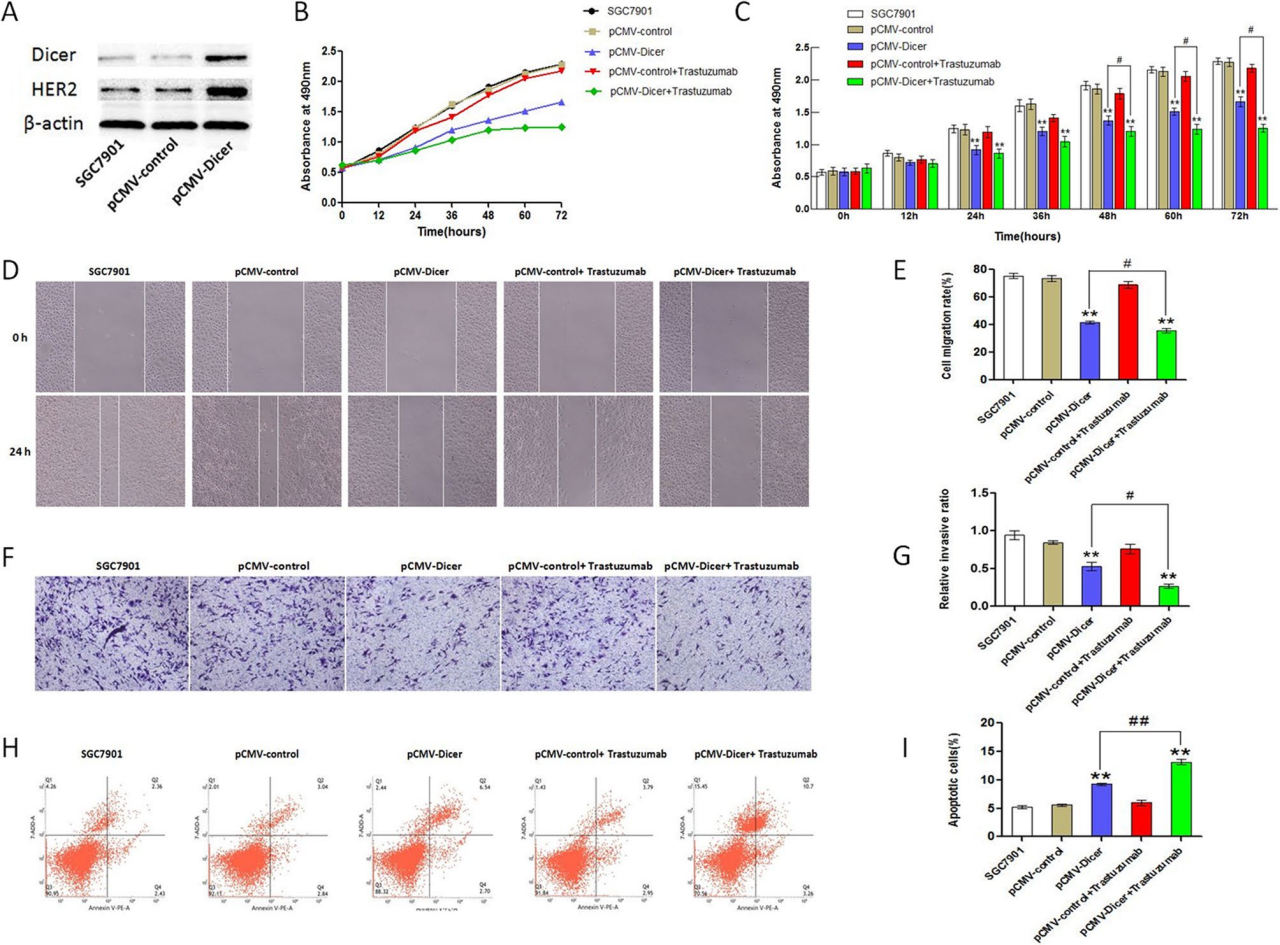
Trastuzumab enhance the Dicer related inhibition for SGC7901 cell. (**A**) Western Blot of Dicer and HER2 in SGC7901 cells. The grouping of gels/blots cropped from diferent parts of the same gel, the full blots are shown in Fig. S4; (**B**, **C**) The SGC7901 cells proliferation measured with CCK-8 assay; (**D**, **E**) The SGC7901 cells migration measured by wound healing assay; (**F**, **G**) The SGC7901 cells invasion measured with transwell assay; (**H**, **I**) The measurement of SGC7901 cells apoptosis rate with flow cytometry. **P < 0.01, ^#^P < 0.05, ^##^P < 0.01.

The protein expression of HER2 and Dicer in GC tissue were semi-quantified by immunostaining with anti-HER2 and anti-Dicer antibody (Table 1 and Supplementary Fig. S1), Dicer is positively correlated with HER2 expression by chi-square test (*p* = 0.002, Table 1 and Supplementary Fig. S1). The interaction between these two genes was evaluated subsequently in GC cell in vivo, as illustrated in Fig. 1A, the HER2 expression was markedly increased upon Dicer overexpression by comparing SGC7901, SGC7901 transfected with pCMV vector and SGC7901 transfected with pCMV-Dicer cells. These data demonstrated that Dicer could mediate HER2 overexpression in GC cell.

**Table 1:** Correlation between dicer protein level and HER2 protein level in GC patients by χ^2^ test.

Protein expression		HER2		χ^2^	p-value
		High	Low		
Dicer	High	10	25	9.206	0.002
	Low	6	75		

HER2 2 + and 3 + were defined as high expression, HER2 0 and 1 + were defined as low expression.

### Dicer inhibited the growth of GC cells

**Figure 2:**
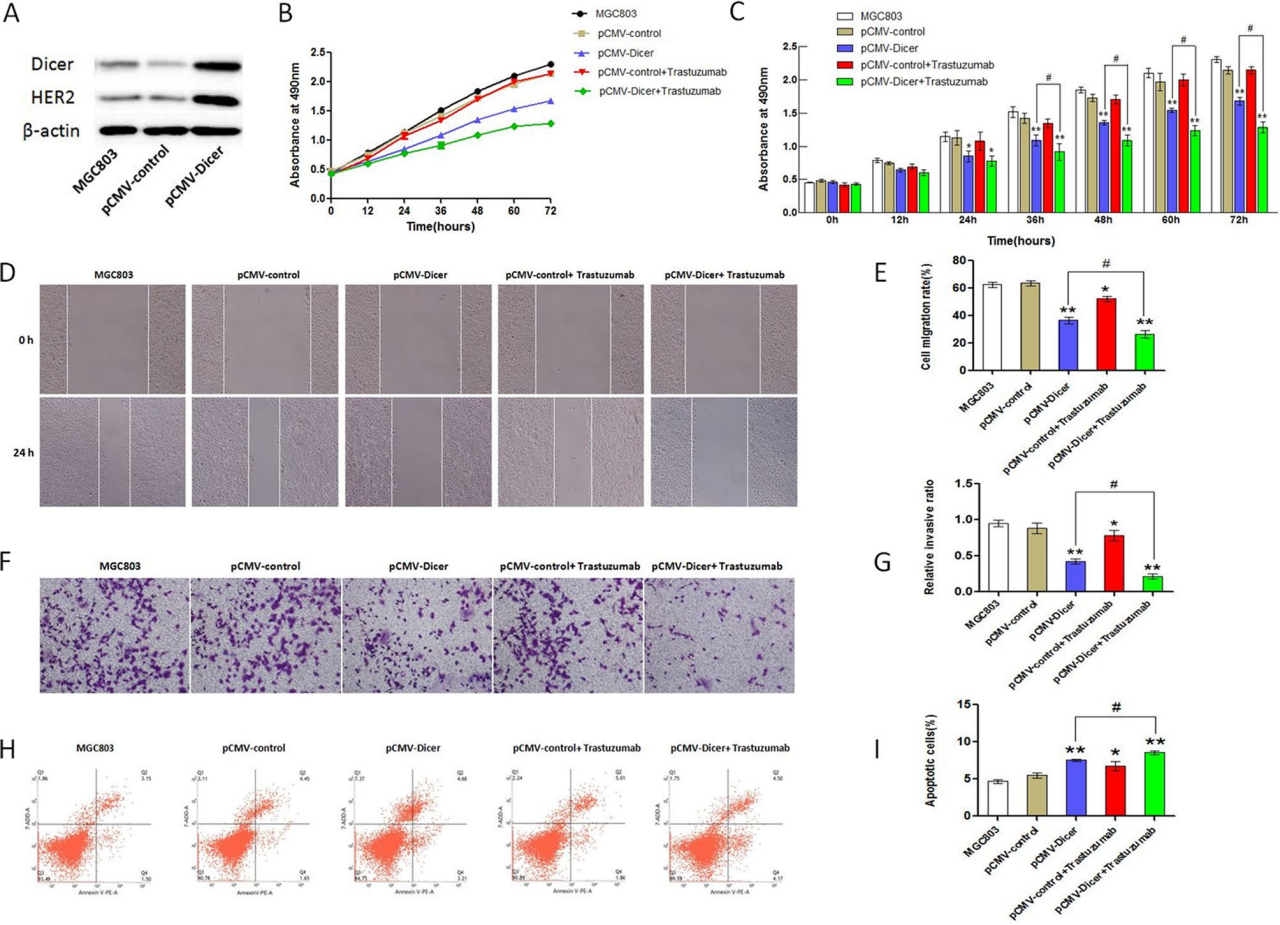
Trastuzumab enhance the Dicer related inhibition for MGC803 cell. (**A**) Western Blot of Dicer and HER2 in MGC803 cells. The grouping of gels/blots cropped from diferent parts of the same gel, the full blots are shown in Fig. S4; (**B**, **C**) The MGC803 cells proliferation measured with CCK-8 assay; (**D**, **E**) The MGC803 cells migration measured by wound healing assay; (**F**, **G**) The MGC803 cells invasion measured with transwell assay; (**H**, **I**) The measurement of apoptosis rate with flow cytometry for MGC803 cells. *P < 0.05, **P < 0.01, ^#^P < 0.05.

The proliferation capacity of SGC7901 cell in three different group including pCMV-Dicer transfected cell, pCMV tranfected cell, and blank control cell was measured with CCK assay at different timepoint, the proliferation capacity of pCMV-Dicer transfected cells was significantly decreased from 24 to 72 h compared with that of pCMV transfected cell and blank control cell (*p* < 0.01, Fig. 1B,C). We subsequently assessed the capacity of migration and invasion for this GC cell upon Dicer overexpression with wound healing assay and transwell assay, as illustrated in Fig. 1D and F. SGC7901 cells transfected with pCMV-Dicer displayed significantly decreased migration capacity than that of control vector and blank control by wound healing assay (*p* < 0.01, Fig. 1E). Moreover, the invasive ratio was significantly decreased in pCMV-Dicer transfected GC cells than that in pCMV transfected GC cells and blank control (Fig. 1G, p < 0.01). To further investigate the mechanism of GC cell inhibition by Dicer overexpression, apoptosis was assessed by Annexin V-PE/7-AAD double staining via flow cytometry. Apoptotic cells were significantly augmented in pCMV-Dicer group compared with those of the control vector and blank control (*p* < 0.01, Fig. 1H,I). These results indicated that Dicer overexpression could inhibit proliferation, migration, and invasion, as well as induce apoptosis for GC cells with high Dicer-induced HER2 expression. We validated our result with another GC cell line of MGC803, and almost the same result was obtained referring to proliferation, migration, invasion, and apoptosis for GC cells (Fig. 2A–I). Furthermore, to confirm that Dicer-induced inhibition for GC was not caused by HER2 overexpression, we overexpressed HER2 in MGC803 cell (Supplementary Fig. S2), and proved that proliferation, migration, and invasion were enhanced by HER2 overexpression.

**Figure 3:**
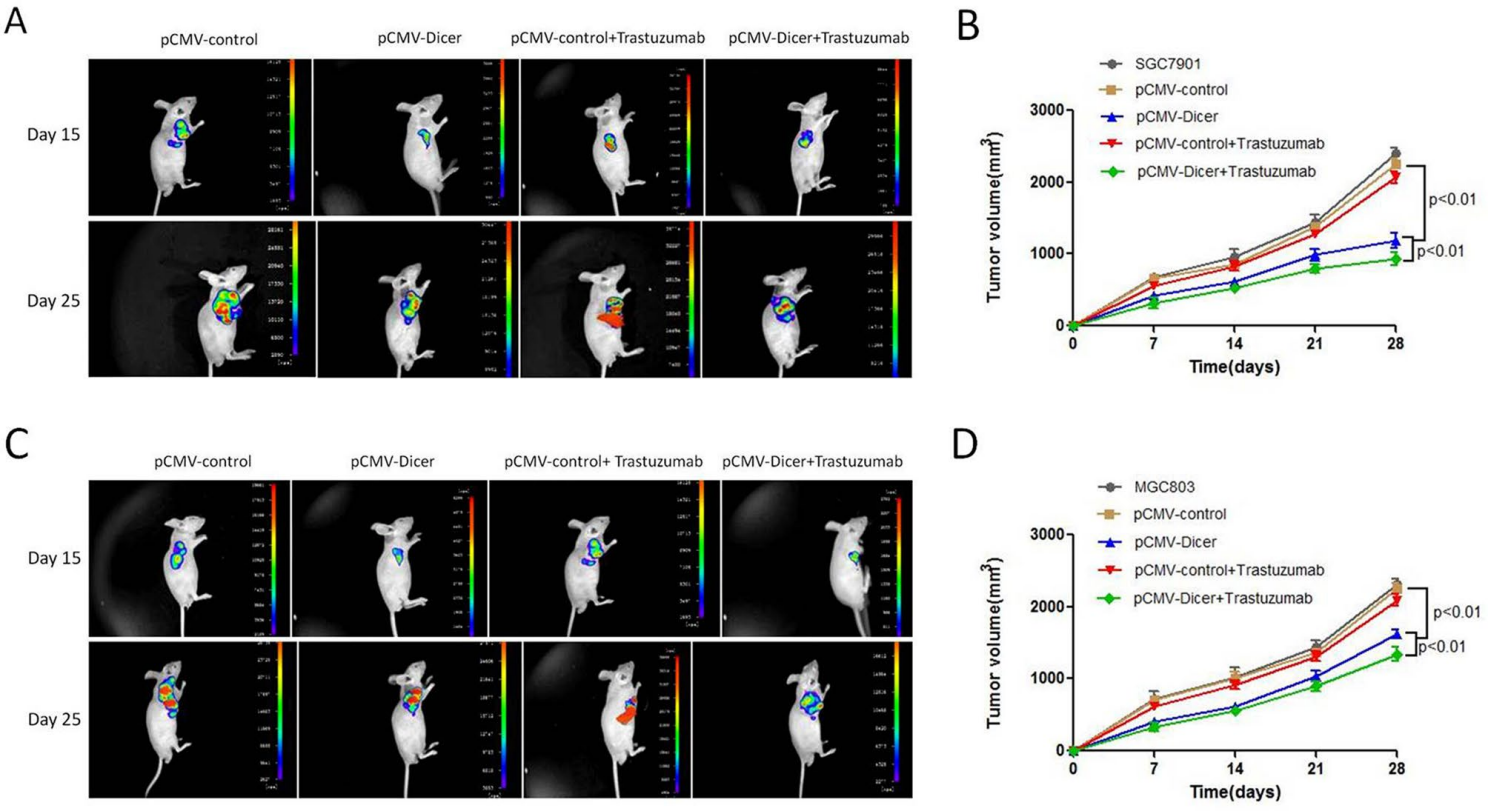
Effects of Dicer overexpression and treatment with Trastuzumab on GC cell growth in vivo. (**A**) Fluorescence image of mice bearing SGC7901 cells xenografts; (**B**) Tumor growth was monitored by measuring tumor volume for SGC7901 xenografts (n = 6 for each group); (**C**) Fluorescence image of mice bearing MGC803 cells xenografts; (**D**) Tumor growth was monitored by measuring tumor volume for MGC803 xenografts (n = 6 for each group).

We evaluated the Dicer initiated GC inhibition in vivo. The growth of Dicer overexpressed xenograft was significantly decreased compared with that of control pCMV xenograft at 25 days after implantation (Fig. 3A), and the tumor volume of Dicer overexpressed xenograft was smaller than that of control xenograft and SGC7901 cell xenograft at 28 days after implantation (Fig. 3B, p < 0.01). Similar result was obtained with MGC803 xenograft transfected with pCMV-Dicer (Fig. 3C,D, p < 0.01). These data demonstrated that the Dicer could inhibit the growth of GC cell with high HER2 expression in vivo.

### Trastuzumab promotes inhibition induced by Dicer for GC cells

Since Dicer could promote HER2 expression in GC cells, we assessed whether the overexpressed HER2 induced by Dicer acts as an efficient target for inhibition by HER2 antibody. The humanized HER2 monoantibody trastuzumab with final concentration of 10 μg/mL was added to the medium incubated with pCMV and pCMV-Dicer GC cells, and subsequently, SGC7901 and MGC803 cells were analyzed for proliferation, apoptosis, migration, and invasion. No difference in proliferation, migration, invasion, and apoptosis was found after trastuzumab addition in SGC7901 cells by comparing control GC cells and GC cell plus trastuzumab (Fig. 1B–I); however, this antibody decreased the proliferation capacity from 48 to 72 h (*p* < 0.05), migration (*p* < 0.05), and invasion (*p* < 0.05), whereas it increased the apoptosis rate (*p* < 0.01) of SGC7901 cell when comparing pCMV-Dicer and pCMV-Dicer plus trastuzumab GC cells (Fig. 1B–I). Trastuzumab incubation could inhibit the migration (*p* < 0.05) and invasion (*p* < 0.05) of MGC803 cell, whereas it increased the apoptosis rate (*p* < 0.05) when comparing MGC803 cell and MGC803 plus trastuzumab cell (Fig. 2D–I). Moreover, trastuzumab inhibited pCMV-Dicer MGC803 cell proliferation from 36 to 72 h (*p* < 0.05), migration (*p* < 0.05), and invasion (*p* < 0.05), as well as promoted apoptosis (*p* < 0.05) by comparing pCMV-Dicer MGC803 cell and pCMV-Dicer plus trastuzumab MGC803 cell (Fig. 2B–I). These data demonstrated that trastuzumab could inhibit the growth of GC cells and amplify GC suppression by Dicer presumably due to the overexpressed HER2 provided effective target for trastuzumab.

Considering xenograft, intraperitoneal injection with 15 mg/kg trastuzumab once every 3 days could not inhibit the growth of pCMV transfected GC cells at any time point (Fig. 3A–D), but the trastuzumab related growth inhibition for pCMV-Dicer-transfected GC xenograft was achieved when compared to pCMV-Dicer and pCMV-Dicer plus Trastuzumab GC cells(*p* < 0.01; Fig. 3A–D). These data demonstrated that trastuzumab injection suppressed at the base of Dicer inhibited GC cells in vivo and in vitro.

### Calcitriol accelerates trastuzumab-related inhibition in GC cells

**Figure 4:**
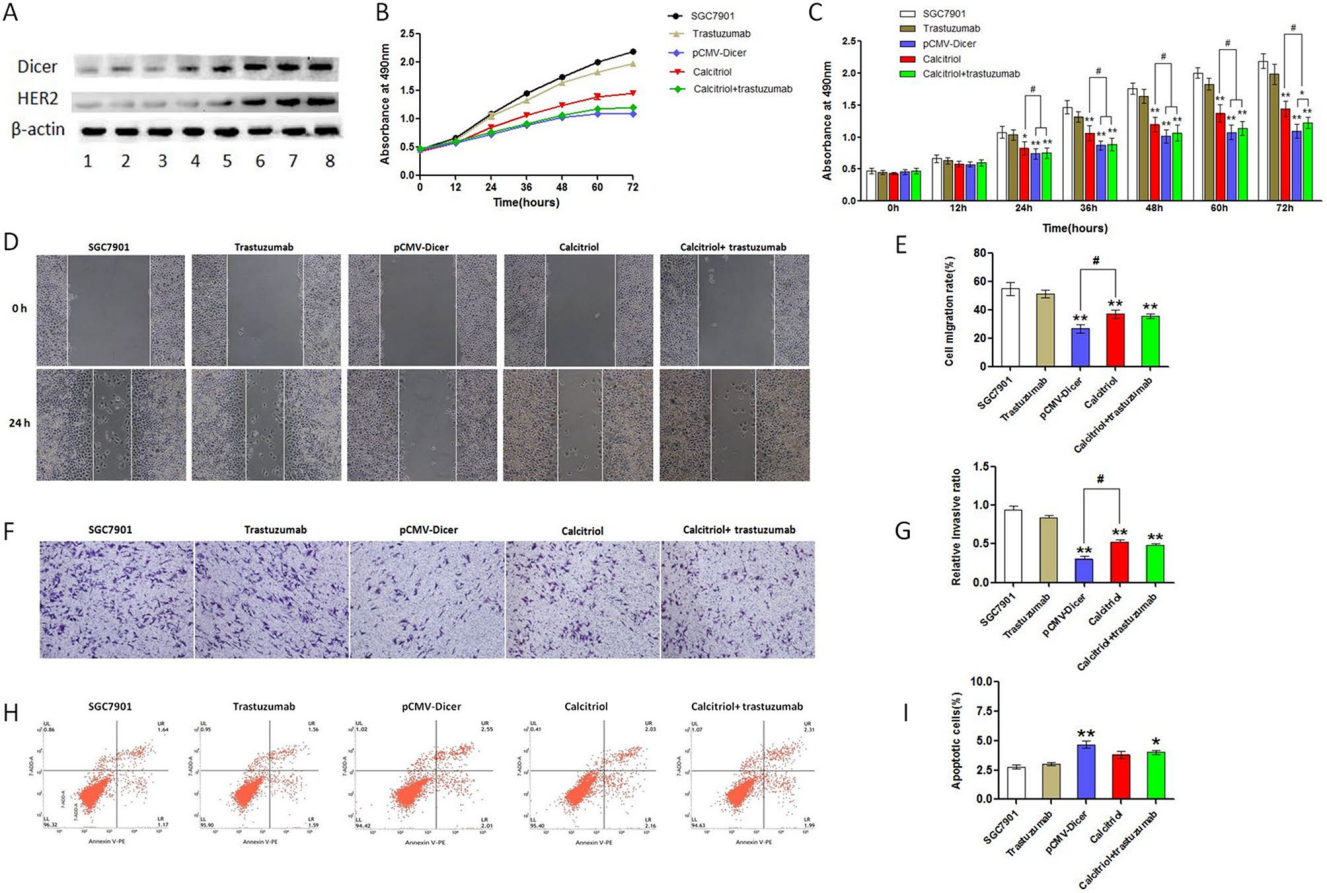
Calcitriol could induce HER2 expression and synergy with Trastuzumab for GC inhibition. (**A**) Western Blot analysis of Dicer and HER2 expression upon Calcitriol induction in SGC7901 cells, Line 1–7 with Calcitriol concentration of 1, 2, 4, 6, 8, 10 and 15 mmol/L, Line 8: pCMV-Dicer. The grouping of gels/blots cropped from diferent parts of the same gel, the full blots are shown in Fig. S4; (**B**, **C**) The SGC7901 cells proliferation measured with CCK-8 assay; (**D**, **E**) The SGC7901 cells migration measured by wound healing assay; (**F**, **G**) The SGC7901 cells invasion measured with transwell assay; (**H**, **I**) The measurement of apoptosis rate with flow cytometry for SGC7901 cells. *P < 0.05, **P < 0.01, ^#^P < 0.05.

Calcitriol can increase the Dicer expression in SiHa cervical cancer cells^24^. We measured the Dicer expression for SGC7901 cells after incubation with calcitriol for 72 h to check whether calcitriol could be used as Dicer inducer in order to initiate HER2 overexpression. As illustrated in Fig. 4A, the concentration of calcitriol in Lines 1–7 is 1, 2, 4, 6, 8, 10, and 15 mmol/L respectively; whereas, Line 8 depicts the concentration of calcitriol in pCMV-Dicer transfected GC cells. The expression of Dicer and HER2 were significantly induced by calcitriol with concentrations of 10 mmol/L and 15 mmol/L; hence, we used the concentration of 15 mmol/L for subsequent inhibition experiment. The single drug treatment with calcitriol inhibited cell proliferation from 24 to 72 h (*p* < 0.05), migration (*p* < 0.01), and invasion (*p* < 0.01) for GC cells at statistically significant levels (Fig. 4B–G), whereas it had no effect on apoptosis (Fig. 4H,I). These data demonstrated that calcitriol induced HER2 overexpression without increasing the malignancy of GC cells; moreover, it could inhibit cell proliferation, invasion, and migration presumably due to Dicer overexpression. The addition of trastuzumab to calcitriol inhibited proliferation of GC cells from 24 to 72 h when compared to the calcitriol and calcitriol plus trastuzumab cells (Fig. 4B,C). Furthermore, the addition of trastuzumab slightly accelerates the inhibition caused by calcitriol referring to migration and invasion for GC cells, but does not reach the statistical levels (Fig. 4D–G). Although no difference was observed for apoptosis between calcitriol and calcitriol plus trastuzumab cells, calcitriol plus trastuzumab increased the apoptosis rate when compared with SGC7901 control cells (Fig. 4H,I, p < 0.05), but calcitriol monotreatment could not achieve apoptosis difference when compared to that of control GC cells. Collectively, calcitriol favored GC cell inhibition induced by trastuzumab by initiating HER2 expression.

## Discussion

We found that Dicer could inhibit the growth of GC cell as well as induce HER2 overexpression without increasing malignancy of GC cell. Moreover, the HER2 overexpression provided more effective and additive target for trastuzumab in inhibiting GC cells. Furthermore, we proved that calcitriol amplified the trastuzumab related GC inhibition by inducing Dicer overexpression to initiate HER2 overexpression. Trastuzumab is an effective first line treatment for GC, a cancer with few effective antiangiogenic drugs due to its heterogeneity, but only 7%–34% patients adapt to trastuzumab, thereby limiting its application in GC patients^3,8,13^. We aim to find an inducer for HER2 overexpression that can help in adapting the low HER2 expressed GC cell to HER2 antibody treatment, simultaneously without increasing the aggressiveness of GC. Dicer is proven to be a suitable inducer based on our data; however, the clinical impracticability of vector transfection hampers its clinical application. Calcitriol induced Dicer expression to promote the efficiency of HER2 antibody in subsequent analysis. Moreover, it amplified the inhibition of trastuzumab to GC cells undergoing proliferation. The suitable concentration for trastuzumab and calcitriol should be calculated to obtain the best inhibition efficiency referring to proliferation. Our finding implied that calcitriol might increase the adabtibility of patients to HER2 antibody via HER2 overexpression; however, this finding needs to be further validated in the model animal for calcitriol referring to HER2 induction and trastuzumab synergism prior to clinical trial. Furthermore, the suitable concentration for calcitriol needs to be evaluated in GC patients for subsequent clinical application. The monotherapy using trastuzumab exhibited only mild inhibition for GC cells based on our data, whereas the combination of chemotherapeutic drugs and calcitriol helped in assessing their synergism for HER2 antibody. Another reagent of Metformin, which can induce Dicer expression in breast cancer cell and affect its growth, will be assessed for its synergism with trastuzumab by inducing Dicer and HER2 expression^32^.

The mechanism by which Dicer expression mediated the overexpression of HER2 without increasing malignancy of GC cell remains unclear. Since Dicer might induce HER2 overexpression via regulating miRNA expression, we performed the miRNA array analysis to identify the potential miRNA that could mediate HER2 expression. Ten miRNAs were upregulated, whereas 24 miRNAs were downregulated with fivefold changes upon Dicer overexpression (Supplementary Fig. S3, Supplementary Table S1). Among these miRNAs, the miR-139 and miR-203a, which were downregulated upon Dicer overexpression, have been proved to be negatively associated with HER2 expression in GC or breast cancer tissue^33–35^. Dicer might induce HER2 expression through downregulating these miRNAs. Further laboratory-based functional studies are warranted to identify these candidate miRNAs in mediating HER2 expression.

In conclusion, the Dicer inducer of calcitriol might be considered as a potential opinion to extend clinical indications of HER2 antibody for low or negative HER2 patients who were not fit for HER2 antibody treatment before.

## Supplementary Information

Supplementary Figures.

Supplementary Table S1.

## Data Availability

Research data supporting the results of this paper will be provided by corresponding author at reasonable request.

## Abbreviations

GC: Gastric cancer
HER2: Human epidermal growth factor receptor 2
VEGF: Vascular endothelial growth factor
AAs: Anti-angiogenic agents
TKIs: Tyrosine kinase inhibitors
GEJ: Gastro-esophageal junction cancer
3′UTR: 3′-Untranslated region
siRNA: Short interfering RNA
SP: Streptavidin-peroxidase
DAB: 3, 3′-Diaminobenzidine
GFP: Green fluorescent protein
CCK-8: Cell counting kit-8
ANOVA: One-way analysis of variance
