# Supplementary material for: Dicer increases the indication for trastuzumab treatment in gastric cancer patients via overexpression of human epidermal growth factor receptor 2
Source: Sci Rep. 2021 Mar 26;11:6993. doi: 10.1038/s41598-021-86485-8 (PMC7997953; doi:10.1038/s41598-021-86485-8)

Dicer increases the indication for trastuzumab treatment in gastric cancer patients via overexpression of human epidermal growth factor receptor 2

Jianhua Wu[1,5], Qun Zhao[2,5], Yue Zhao[3], Xiaoyun Zhang[4], Yuan Tian[2], Zhanjun Guo[4,] *

[1]Animal Center, [2]Department of Surgery, [3]Department of Gastroenterology and Hepatology, [4]Department of Immunology and Rheumatology, The Fourth Hospital of Hebei Medical University, Shijiazhuang, P.R. China.

[5]These authors contribute equally to this work.

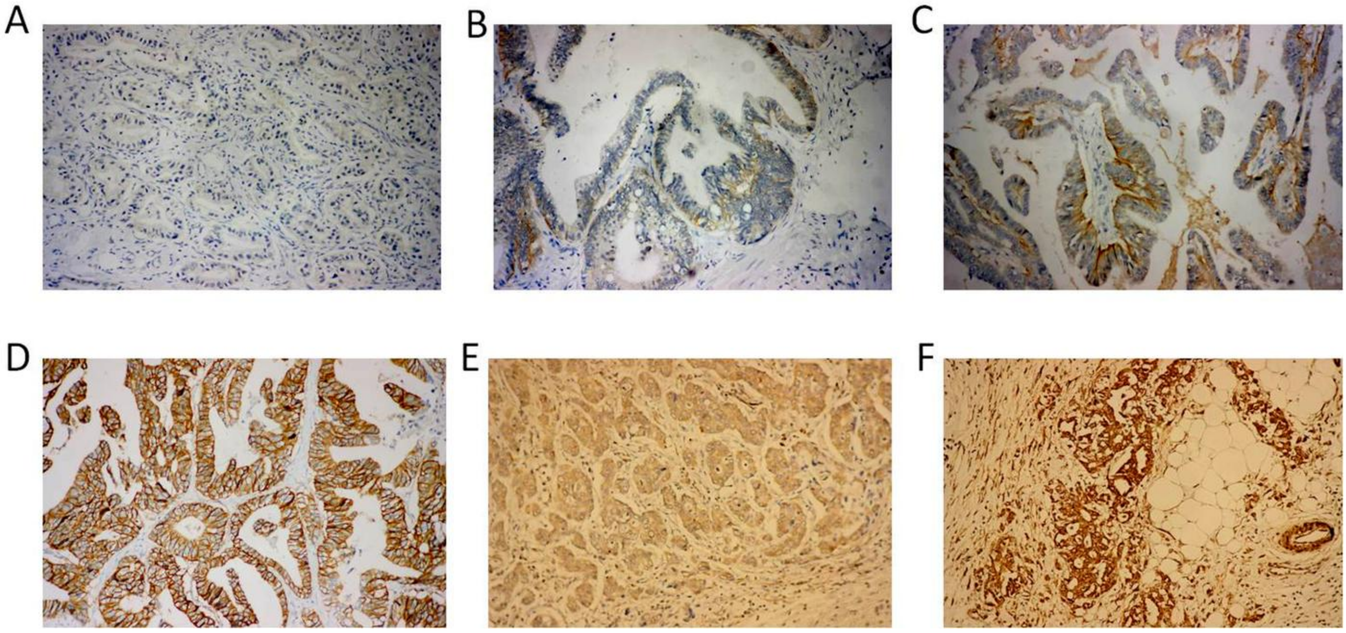

Figure S1. Immunostaining of Dicer and HER2 in GC tissues.

(A) The HER2 expressional level of “0”; **(B)** The HER2 expressional level of “1+”; **(C)** The HER2 expressional level of “2+”; **(D)** The HER2 expressional level of “3+”; **(E)** The low expression of Dicer; **(F)** The high expression of Dicer. Original magnification, ×200.

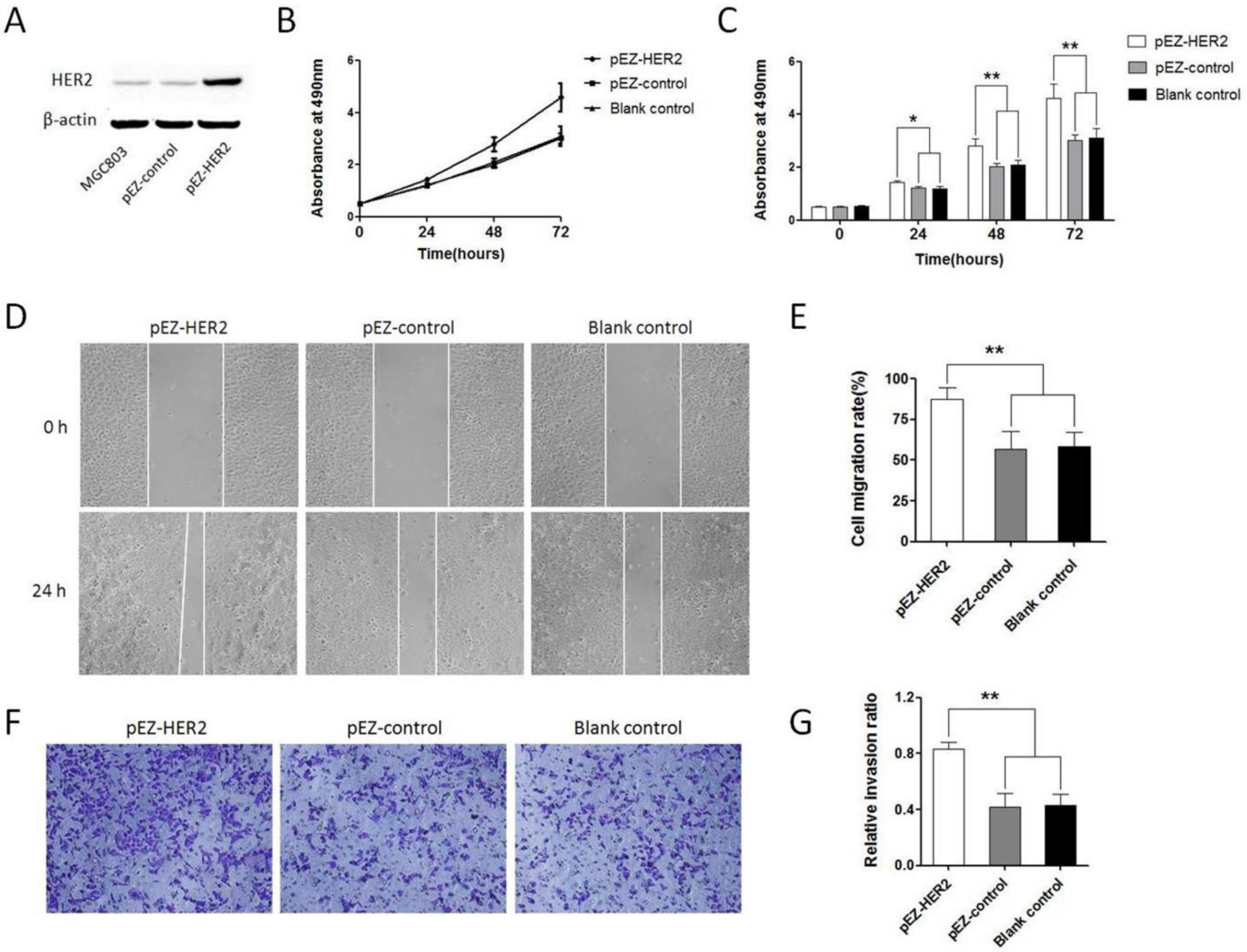

Figure S2. HER2 overexpression promotes proliferation, migration, and invasion of GC cells.

(A) Western Blot of HER2 protein in MGC803 cells transfected with pEZ-HER2 or pEZ-control plasmids. The grouping of gels/blots cropped from diferent parts of the same gel, the full blots are shown in Figure S4; **(B, C)** The MGC803 cells proliferation measured with CCK-8 assay; **(D, E)** The MGC803 cells migration measured by wound healing assay; **(F, G)** The MGC803 cells invasion measured with transwell assay. *P<0.05, **P<0.01.

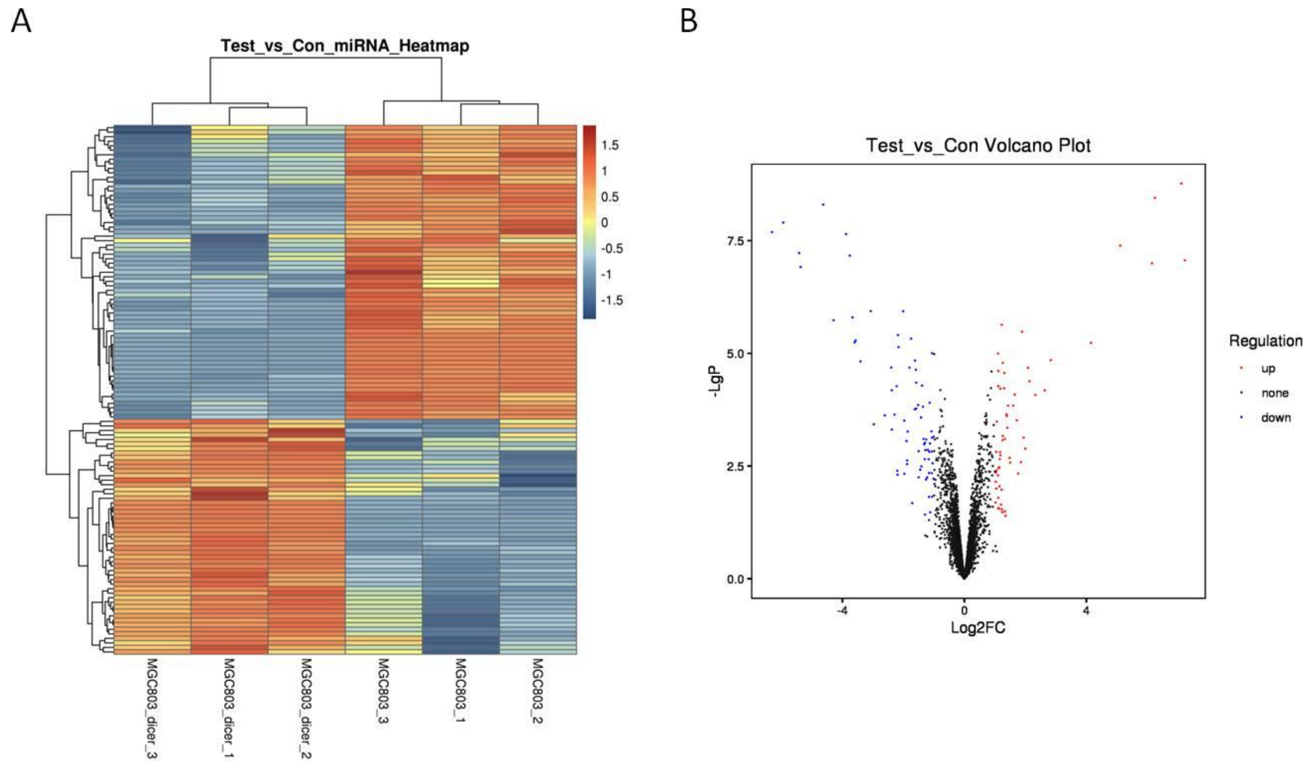

Figure S3. miRNA expression profile differences between the Dicer overexpression group and the control group in MGC803 cell.

(A) Hierarchical Cluster analysis of differentially expressed miRNAs between the Dicer overexpression group and the control group. **(B)** Volcano plot shows significant differences between the Dicer overexpression group and the control group. Red dot, up-regulated >2 fold and p<0.05; blue dot, down-regulated >2 fold and p<0.05.

Figure S4 The grouping of gels/blots cropped from diferent parts of the same gel.

Figure 1A

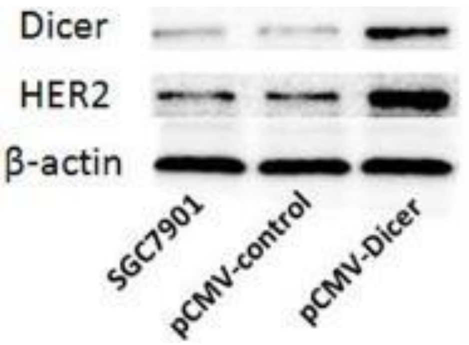

Original gels for Figure 1A

Dicer

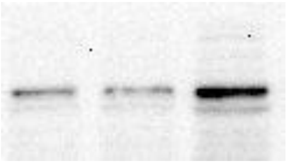

HER2

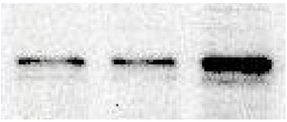

β-actin

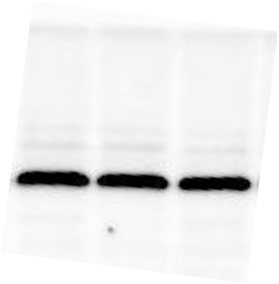

Figure 2A

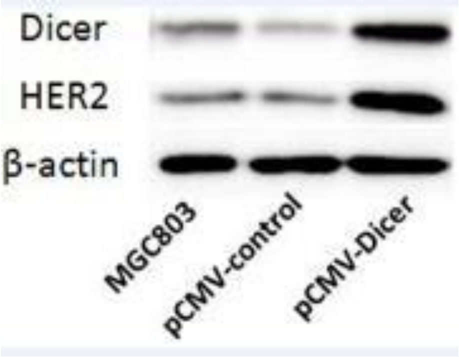

Original gels for Figure 2A

Dicer 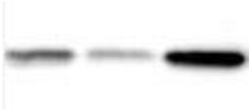

HER2 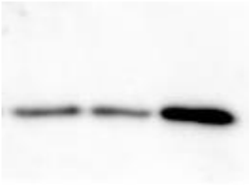

β-actin 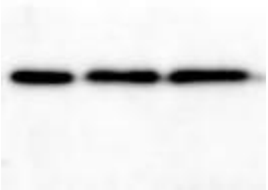

Figure 4A

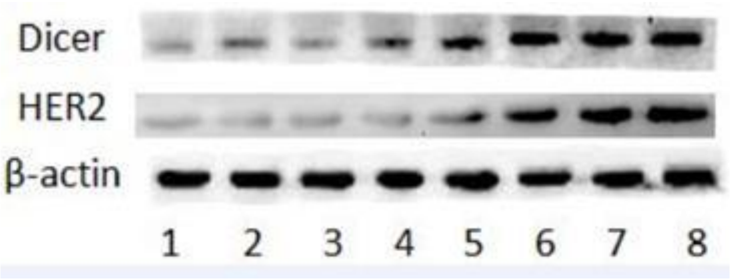

Original gels for Figure 4A

Dicer

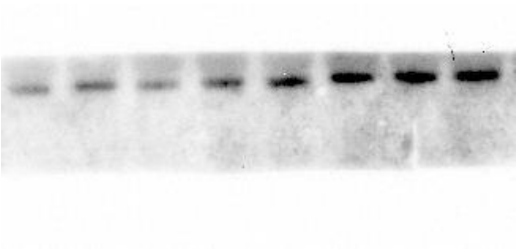

HER2

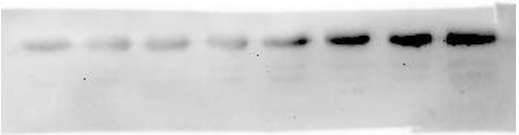

β-actin

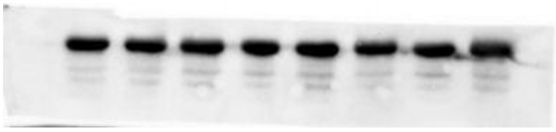

Figure S2

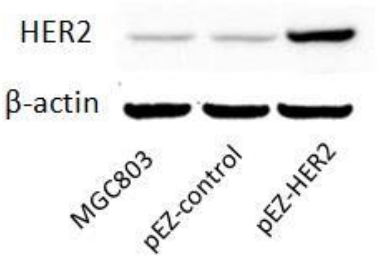

Original gels for Figure S2

HER2

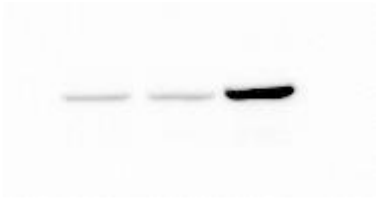

β-actin

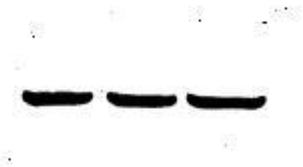

Supplement: Supplementary file 1 — Supplementary Figures. [file 41598_2021_86485_MOESM1_ESM.pdf]
